# Osteoporosis in children: pediatric and pediatric rheumatology perspective: a review

**DOI:** 10.1186/1546-0096-7-16

**Published:** 2009-10-16

**Authors:** Yosef Uziel, Eyal Zifman, Philip J Hashkes

**Affiliations:** 1 Pediatric Rheumatology Unit, Pediatric Department, Meir Medical Center, Kfar Saba, Israel; 2 Sackler School of Medicine, Tel Aviv University, Tel Aviv, Israel; 3 Section of Pediatric Rheumatology, Dept of Rheumatic Diseases, Cleveland Clinic Foundation, Cleveland OH, USA

## Abstract

It is increasingly recognized that osteoporosis affects children as well as adults both as a primary problem and as secondary to various diseases, medications, and lifestyle issues. In this review, we emphasize the correct diagnosis of osteoporosis in children as opposed to adults, etiology, and pharmaceutical and non-pharmaceutical treatments. We especially focus on rheumatologic conditions associated with osteoporosis and management issues.

## Introductory case 1

An 11-year-old, previously healthy girl presented with a new fracture of the femoral bone following a mild sports injury. Plain x-ray revealed an osteoporotic fracture. What is the cause? What therapy is recommended for preventing new fractures?

## Introductory case 2

**Figure 1:**
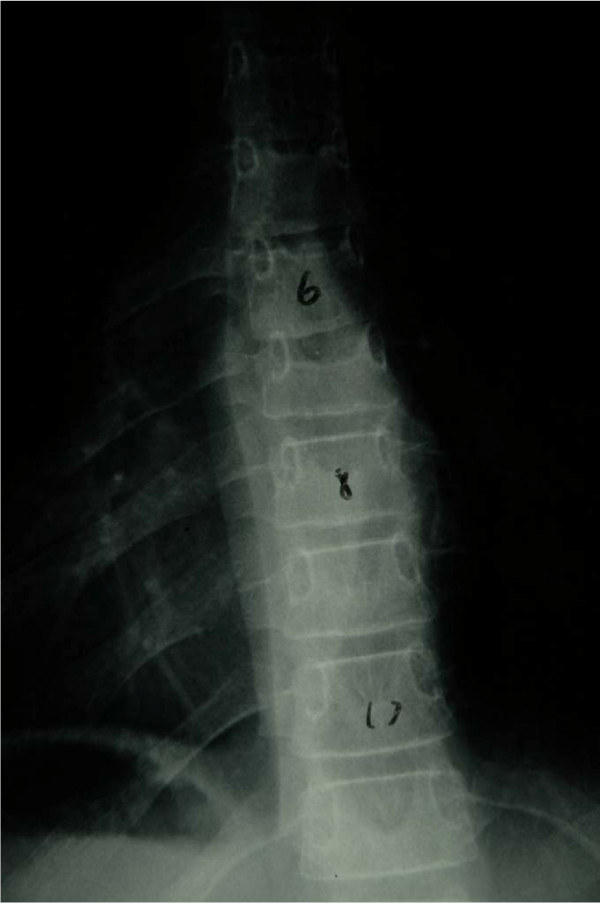
**Spine radiograph demonstrating an osteoporosis-related T7 vertebral fracture**.

A 13-year-old boy was treated with corticosteroids for juvenile idiopathic arthritis. Which therapy might be helpful for reduction of osteoporosis? Later he developed back pain and an osteoporotic fracture in the T7 vertebra (Figure 1). Which therapy is suggested to prevent additional fractures?

## Background

There is greater awareness and recognition of osteoporosis (OP) in children both as a primary problem and as a harbinger of OP in adulthood. Although it was once thought to afflict mainly adults with chronic diseases and elderly patients, in recent years OP has been recognized as a pediatric problem as well.

In this article, we will review the common forms of pediatric OP and emphasize the latest treatment data available in the literature.

### Definition and measuring osteoporosis

In adults, OP is commonly defined as a bone density of 2.5 standard deviations (SD) below the mean in dual emission x-ray absorptiometry (DXA) [1]. Osteopenia is defined as a bone density between 1.0 and 2.5 SD below the young adult mean. In the pediatric population, a somewhat different definition exists, requiring both a history of pathologic fractures and low bone mineral content or density [2]. These criteria are fulfilled by the diagnosis of a single significant fracture in a long bone of the lower extremity, two fractures in the long bone of an upper extremity, or one vertebral compression fracture.

Bone density varies greatly with age. This is the reason the densitometry Z-score is used in the pediatric population and not the T-score usually used in adults. Z scores of -2 SD define OP [3].

The problem with pediatric DXA studies is the over-diagnosis of OP in children due to misinterpretation of data based on adult references [4]. To help correct this misinterpretation, the Stanford reference graph was created based on an evaluation of 423 healthy children from various ethnicities. It is currently used for children by converting DXA data adjusted for age and sex .

Other modes of assessing OP are ultrasound (US) by measuring the bone speed of sound (SOS) at the tibial, radial, or calcaneal bones reflecting both cortical density and thickness [5-7] or quantitative computed tomography (QCT) which can supplement DXA by enabling geometric and compartmental bone assessments [8,9]. While US is being used more frequently in pediatrics, QCT is less known and DXA is the still considered gold standard for assessing OP.

Common laboratory tests for evaluation of OP include calcium, phosphate, bone alkaline phosphatase, vitamin D levels (most common vitamin D25), parathyroid hormone levels, and 24-hour urinary calcium and phosphate. More specific bone turnover markers include serum PINP (propeptide of type I procollagen) and carboxyterminal (PICP) propeptides of type I collagen as bone formation marker, and amino-terminal (NTX) and carboxyterminal (CTX) telopeptides of type I collagen as bone resorption markers.

### Etiology

There are many possible causes for OP in children - most are secondary to other illnesses or treatments, especially glucocorticoid (GC) use (Table 1). The most common primary bone disorder leading to OP is osteogenesis imperfecta (OI), a structural genetic defect in the quantity or quality of bone type 1 collagen production. OI has several subtypes ranging from mild forms to that which causes intrauterine fetal death. The family history, the blue, purple, or gray sclera commonly found in OI, radiographic findings, and in some cases, bone biopsy help determine the diagnosis.

**Table 1:** Etiology of Osteoporosis in Children.

**Primary bone disorders**	**Chronic inflammatory diseases**
Osteogenesis imperfecta	Systemic lupus erythematosus

Idiopathic juvenile osteoporosis	Juvenile idiopathic arthritis

Osteoporosis-Pseudoglioma syndrome	Dermatomyositis

Homocystinuria	Inflammatory bowel disease

Ehlers-Danlos syndrome (type I)	Nephrotic syndrome

Marfan syndrome	**Immobility or decreased activity**

GSD type 1	Post trauma

Juvenile/Early-onset Paget's disease	Cerebral palsy

**Catabolic state/Inadequate nutrition/Malabsorption**	Spinal muscular atrophy, Muscular dystrophy

Vitamin D deficiency	**Medications**

Malignancy - Acute lymphoblastic leukemia, Lymphoma	Anticonvulsant, Glucocorticosteroids, Heparin, Methotrexate (in oncology doses)

Cystic fibrosis	**Endocrine disorders**

Psychiatric eating disorders - Anorexia nervosa/Bulimia	Hypogonadism - Gonadal dysgenesis

Chronic malabsorption (e.g. Celiac disease)	Hyperthyroidism

Acquired immunodeficiency syndrome	Cushing syndrome

Female athlete triad disorder	Growth hormone deficiency

	Delayed puberty

Other primary bone causes of OP include idiopathic juvenile osteoporosis (IJO) [10], and osteoporosis-pseudoglioma syndrome; these two are rarely encountered.

Secondary causes of OP are much more common. There are several subgroups, including systemic inflammatory diseases which affect bone metabolism due to high inflammatory cytokine levels and corticosteroid use and endocrine disorders such as diabetes mellitus, hyperparathyroidism, hyperthyroidism, hypercortisolism, and hypogonadism that act via altered hormone levels. Any cause of immobilization, such as in cerebral palsy or spinal muscular atrophy, decreases bone density due to diminished mechanical stimulation. Also important to note are neoplastic disorders (especially leukemia and lymphoma), medications such as GC, anticonvulsants, and heparin. Finally, decreased dietary intake of calcium, vitamin D deficiency, malabsorption, and a low calorie diet in endurance athletes are additional risk factors for OP.

Discoveries in recent years have disclosed other aspects of bone turnover. The receptor activator of nuclear factor κB (RANK) and its ligand (RANKL), along with another cellular moiety - osteoprotegerin (OPG) - were found to be important participants in osteoclast differentiation and proliferation. While the activation of RANK with its ligand molecule promotes osteoclast proliferation, the RANK receptor OPG actually inhibits this process, subsequently decreasing osteoclast activity and bone resorption. Mutations in the OPG and RANK genes are responsible for two hereditary forms of primary bone disorder, namely juvenile Paget's disease and early-onset Paget's disease [11].

However, the RANK-RANKL-OPG system is also involved in bone resorption due to systemic GC use. Administration of GC induces synthesis of RANKL and inhibits production of OPG [12]. This may be the reason for osteopenia and OP found after chronic use of these drugs. GC also inhibit replication, differentiation and function of osteoblasts and induce the apoptosis of mature osteoblasts and may inhibit bone building functions or calcium absorption through nongenomic effects, for example by interactions with biological membranes, either through binding to membrane receptors or by physicochemical interactions.

### Systemic lupus erythematosus, juvenile idiopathic arthritis and dermatomyositis

Individuals with juvenile onset SLE are more prone to suffer from decreased bone mineral density (BMD) and hence OP [13,14]. Factors recognized to be responsible for these effects are various cytokines and GC use, especially via the RANK-RANKL-OPG system. Compeyrot-Lacassagne et al. [15] described prevalence values for osteopenia and OP among juvenile SLE patients. In their cohort, rates for osteopenia and OP were 37.5% and 20.3%, respectively. Factors associated with these conditions were disease duration, use of GC and cytotoxic drugs and disease severity, especially nephritis. Reported OP rates were slightly lower at 12.1% in a large cohort of 1000 adult patients followed for 10 years [16]. Similar findings have been reported for dermatomyositis [17]. While not yet proven in comparative studies, it is the impression of many clinicians that the OP of dermatomyositis may be more severe than other autoimmune diseases even when treated with the same type and dose of glucocorticoids. Perhaps the relatively greater immobilization in myositis may play a role, as well as a more prolonged/higher cumulative dose.

A study by Aggarwal et al. [18] found decreased BMD in adult patients previously diagnosed with JIA. Likewise, Okumus et al. [19] found lower BMD levels in 30 JIA patients, especially in the polyarticular group. While they did not detect a statistically significant relationship with disease duration, BMD and Z score were associated with lower insulin-like growth factor I (IGF-I) levels. Henderson et al. [20,21] described low total body BMD (Z score lower than -1SD) in 29.2% of prepubertal and postpubertal JIA patients. Notably, none of the JIA patients or controls had ever received GC. As noted, those with greater disease severity and inflammatory markers were more prone to have low BMD. Hartman et al. also demonstrated osteoporosis by DXA and by ultrasound in children with chronic rheumatic diseases [5]. It is important to note that interleukin-6, an important cytokine in JIA, especially systemic JIA, may be an important factor in the development of OP by stimulating osteoclast activity, as demonstrated in an interleukin-6 transgenic mice model by De Benedetti et al. [22]

Clinically, OP may present with a painful bone fracture often associated with mild trauma, whether in a child with a chronic disease such as JIA, or in a previously healthy child with a mild form of OI.

Osteoporosis in the pediatric age group carries little mortality; however, it does bring a considerable burden of morbidity, especially due to pain, interference with regular activities, and long-term sequele.

### Treatment

While the guidelines for the treatment of OP in adults are widely accepted, the much less abundant data for children and adolescents with OP makes it harder to set clear guidelines for the pediatric population.

### Physical activity

It is currently acknowledged that the main factor for achieving peak bone mass in any healthy individual is genetically determined [23]. However, during the last two decades, the notion that weight bearing physical activity (WBPA) during childhood and puberty affects bone mass and formation has become widely accepted. This critical period in life affects bone health during later stages and may promote or inhibit one's tendency to develop osteopenia and OP. Research on the effect of WBPA on bone mineral density (BMD) in children and adolescents has been on the rise for several years.

Rideout et al. [24] have shown that increased WBPA during 12-18 years of age was directly correlated with BMD and other factors examined in both lumbar vertebra and the femur necks of healthy post-menopausal women. Activity level at other ages was found to be much less significant.

It may not be surprising that overall decreased physical activity was found to be associated with decreased BMD in a group of hemophilic children [25]. Likewise, Ausili [26] reported decreased BMD in children with myelomeningocele who were confined to a wheelchair compared to those who were able to walk. In both groups, those who participated in regular physical activity had higher BMD levels than those who did not.

Lien et al. reported that when compared with healthy controls, patients with early polyarthritis had low bone mineral content and low mineral contact gain that was significantly associated with the level of weight bearing exercise [27]. Alwis et al. [28] reported the effects of increased physical education classes on lumbar vertebral and femoral neck characteristics among healthy first and second graders. Those participating in the study's interventional group had 3.3 times the amount of physical activity as the control children. Using DXA, they found that although no statistically significant changes were found in the femoral neck, the width and bone mineral content (BMC) of the L3 vertebra were increased in the children who had more physical education classes. Similarly, in a 7-year follow-up study of 142 adolescent girls, increased physical activity was associated with greater lumbar vertebral and femoral neck BMC. Although the increase was modest, it was statistically significant [29].

The effect of activity on bone health also depends on nutrition. Chevalley et al. [30] demonstrated that with the same amount of energy expenditure at various physical activities, prepubertal boys who consumed more protein had a greater increase in BMD.

### Calcium and Vitamin D

Daily oral intake of calcium is important for maintaining adequate homeostasis and facilitating bone remodeling and growth. The daily recommended intake (RDI) of calcium changes with age, from 500 mg at 1-3 years, to 800 mg for 4 to 8 year-olds, and 1300 mg for 9-18 years of age [31]. Many children and adolescents do not ingest enough calcium to meet the RDI. Those at risk of OP should receive calcium supplements to insure adequate intake.

In a meta-analysis performed by Winzenberg et al. [32] little change in BMD was detected after calcium supplementation in healthy children. Still, patients with underlying disorders affecting bone metabolism may receive greater benefit from such interventions, as Carrasco et al. [33] and Lovell et al. [34] have both demonstrated. By performing two separate, double blind, randomized controlled trials, they were able to demonstrate a decrease in bone turnover markers in JIA patients given supplemental calcium compared to placebo. However, the increase of BMD in the study of Lovell et al., was only slightly greater than with placebo. In the study of Carrasco et al, vitamin D was also provided for both the intervention and the control groups. A simpler design utilizing increased dietary calcium intake conducted by Stark et al. [35] found increased BMC over a 6-12 month period in JIA patients aged 4-10 years.

The intake of appropriate levels of calcium alone may not be enough. Evidently, the composition of the entire diet is important for calcium utilization as shown by Seiquer et al. [36]. They found that a Mediterranean-type diet improved absorption and reduced secretion of ingested calcium

Likewise, vitamin D is crucial not only for bone health and maintaining serum calcium and phosphate levels, but may also be involved in promoting innate immunity [37]. Vitamin D plasma levels in the general population have been decreasing in recent years in response to reduced sun exposure and outdoor activities, increased use of sunscreen and less exercise. The RDI for vitamin D was recently increased to 400 IU [38].

Bowden et al. [39] recently reported decreased levels of 25-hydroxyvitamin D levels among a large group of pediatric patients with OP and osteopenia. Although no direct connection with fracture risk was detected, they surmised that vitamin D supplementation in children with osteopenia and OP is advisable and may indeed reduce morbidity.

Similar findings were described in a follow-up study of 34 pediatric patients with acute lymphoblastic leukemia (ALL) [40]. Although mean bone density was shown to increase one year after therapy cessation, 1,25-dihydroxyvitamin D levels were lower among those suffering from fractures compared to ALL patients without fractures during the follow-up period.

Both Okumus et al. [19] and Bianchi et al. [41] found low vitamin D levels in JIA patients treated with GC. They also reported decreased BMD levels, resulting from high parathyroid hormone levels - the direct outcome of vitamin D deficiency.

The recommended daily consumption of vitamin D may not be sufficient for patients undergoing bone-affecting treatments and those suffering from primary bone disorders. Maalouf et al. [42] demonstrated that high-dose vitamin D supplementation is safe. The efficacy of such a regimen (manifested as higher levels of vitamin D and it's metabolites in serum) was demonstrated by Kilpinen-Loisa et al. [43]. Such therapy may augment bone density and help prevent fractures and related morbidity.

The minimum blood levels of 25OH-vitamin D recommended for patients with OP and patients receiving corticosteroids are 32 ng/ml or per some experts even 40 ng/ml [44]. El-Hajj et al. [45] demonstrated that a high dose of 2000 IU/day of vitamin D supplementation for one year increased lean mass, bone area, and bone mass in children.

### Bisphosphonates

Bisphosphonates are a group of molecules analogous to inorganic pyrophosphate. Unlike pyrophosphate, bisphosphonates are resistant to normal enzymatic breakdown within human tissues. They concentrate within bones, binding specifically to its inorganic components. Bisphosphonates also enter osteoclasts and inhibit their bone destruction ability via several cellular mechanisms. The newer bisphosphonates, synthesized over the last couple of decades, are much more effective than their predecessors and affect bone growth to a much lesser degree, making them more suitable for use in children and adolescents. They are also pain modulators and have anti-inflammatory properties through regulation of inflammatory cytokines including TNF, IL-1, and IL-6.

Bisphosphonate therapy over a long duration seem to be well-tolerated and safe according to two recent reviews by Ward et al. [46] and Bachrach et al. [47] that included a meta-analysis. Moreover, there seemed to be a consistent benefit in terms of increased BMD among the trials inspected.

Intravenous pamidronate appears to be useful for providing immediate pain relief resulting from fractures, mainly vertebral, but its effectiveness in further decreasing the fracture rate has not been proven; most data are from OI treatment.

Poyrazoglu et al. [48] published their experience with 35 pediatric OI patients treated with IV pamidronate. After treatment, these children had increased BMD scores, decreased bone turnover according to lower bone turnover markers, and decreased fracture rates. It was also concluded that earlier age at onset of therapy was related to better response and higher BMD readings. Growth was not stunted by the bisphosphonate therapy. Moreover, besides fever during the first treatment cycle, no other significant adverse events occurred, making this a safe regimen.

Rauch et al. [49] conducted a double-blind placebo-controlled study with oral risedronate for mild OI. After two years of treatment, serum bone resorption marker levels decreased and BMD increased significantly compared to the control group.

Streeten et al. [50] reported nine new cases of OP pseudoglioma (OPPG), a rare autosomal recessive disorder resulting in congenital blindness and evolving OP. In their article, four patients were receiving bisphosphonates regularly with good response, as surmised from serial BMD testing. Barros et al. [51] reported similar findings in two brothers with OPPG treated for four years. They also described a decrease in fracture events, citing the higher BMD as clinically significant.

Apkon et al. [52] found a mean increase of 12-28% in BMD at three different locations in three boys with muscular dystrophy, treated with weekly oral alendronate. Two of them were wheel-chair bound. Despite the small cohort of examined patients, the study emphasized the potential of such interventions.

Children who receive corticosteroids are at increased fracture risk. Inoue et al. [53] performed a prospective, controlled study with five children suffering from autoimmune diseases. All participants had GC-induced OP. Their therapeutic regimen consisted of IV alendronate therapy at 3-month intervals for a total of two years in the intervention group. Although femoral neck BMD decreased over the period of the study, in those patients receiving alendronate, it actually increased. They also noted a decrease in serum bone turnover markers tested, that correlated with the improvement in BMD. No adverse affects were reported during bisphosphonate therapy.

There are various regimens: the dose of pamidronate is 1-3 mg/kg/day once a month to once every 3-4 months, to be used for severe pain from fractures. Usually there is dramatic pain relief. The outcome relating to fracture prevention is not yet established. The optimal duration of oral or intravenous therapy is not known. Acute mild adverse effects include malaise, myalgia, bone pain, diarrhea, and fever; all are transient and self-limited. Hypocalcemia, hypomagnesemia, and hypophosphatemia are far less common. Other uncommon side effects, like esophagitis, have been reported [47]. Radiographs frequently demonstrate "growth-lines" that have no clinical significance.

For primary prevention, use of bisphosphonates in children receiving corticosteroids for chronic disease is limited and currently is not recommended [46].

A group of 15 ALL pediatric patients treated with high dose GC regimens and diagnosed with OP participated in a bisphosphonate therapy study conducted by Lethaby et al. [54]. The duration of treatment ranged from 6 to 24 months by the end of which 14 of the 15 children had improvement in their BMD Z-scores. The remaining patient's score did not change. They also showed that alendronate use did not alter height velocity, regardless of therapy duration.

What happens to drug availability after therapy stops? While bisphosphonates are rapidly cleared from the blood (half-life of several hours), in bones they have a substantially longer half-life, measured in years. Papapoulos and Cremers [55] reported urinary excretion of bisphosphonates in seven patients at a mean duration of 7.7 years after treatment was discontinued. This should be taken into consideration when prescribing those drugs to teenage girls, for the effect on fetal development has not yet been fully determined. Existing data, although very limited, do not suggest any adverse outcomes [56]. It is possible that there are differences between various types of bisphosphonates in regard to bone half-life, but that has not been studied in young females.

Apart from that, it was shown by Waterhouse et al. [57] that the beneficial effects are sustained well beyond the course of treatment. In their follow-up, with a mean duration of 26 months, they found increased BMD (especially vertebral), decreased fracture rates, and no compromise of growth or kidney function.

Bianchi et al. [58] conducted a long term (up to 10 years) follow-up study of 43 children who were treated with alendronate for connective tissue disease (most were treated with corticosteroids for a prolonged time). They concluded that alendronate was safe, seemed to reduce the risk of fractures, and increased Z-scores. It appears that the effect of alendronate on BMD is independent of the connective tissue disease activity [59], and that alendronate may improve height gain velocity and bone mechanical strength even when patients are treated with GC [60].

A possible major side effect of bisphosphonate is osteonecrosis of the jaw, which was reported only in adults [61,62]. Therefore, pre-assessment by a dental surgeon is suggested for evaluation of wisdom tooth extraction and delaying dental treatment until bisphosphonate administration is stopped. A special consideration in adolescence is orthodontic dental work.

### Parathyroid hormone

A few years ago, the Food and Drug Administration (FDA) approved the use of recombinant human parathyroid hormone (rhPTH) for the treatment of OP. However, several concerns have risen regarding the safety of such an agent, especially in younger patients, owing to reports of the development of bone tumors in rats [63]. Still, the connection is not absolutely clear [64] and ongoing research supplies newer data which suggest a smaller risk of primary bone tumors from rhPTH use [65]. Extensive work is yet to be done before rhPTH is part of the regular treatment for OP in children.

In conclusion, there are still uncertainties regarding bisphosphonate use in pediatric OP. Its effectiveness regarding fracture prevention and length of maximal bone mass gain, as well as its long-term effects and safety are unknown.

Regarding the management of the introductory cases: for case #1, physical therapy, enriched diet, and increased calcium and vitamin D, with no use of bisphosphonates is the preferred regimen. For case #2, treatment should be the same, with the possible addition of bisphosphonates. In severe cases of painful fractures, the use of bisphosphonates is justified.

An osteoporotic child has to be treated with a team approach, including a lead pediatrician, a pediatric rheumatologist or endocrinologist who is familiar with OP management, together with a radiologist, orthopedic surgeon, physiotherapist, dietician, psychologist, and nurse. The goal is to improve the child's bone health, which has lifelong implications.

## Abbreviations

(OP): Osteoporosis; (DXA): Dual emission x-ray absorptiometry; (OI): Osteogenesis imperfecta; (GC): Glucocorticoid; (SLE): Systemic lupus erythematosus; (JIA): Juvenile idiopathic arthritis; (WBPA): Weight bearing physical activity; (BMD): Bone mineral density; (BMC): Bone mineral content; (ALL): Acute lymphoblastic leukemia; (QCT): Quantitative computed tomography; (US): Ultrasound

## Competing interests

The authors declare that they have no competing interests.

## Authors' contributions

YU participated in literature review and writing the manuscript. EZ participated in literature review and writing the manuscript. PJH participated in literature review and writing the manuscript
